# Variability in competitive fitness among environmental and clinical azole-resistant *Aspergillus fumigatus* isolates

**DOI:** 10.1128/mbio.00263-24

**Published:** 2024-02-26

**Authors:** Shu Chen, Guoxing Zhu, Huiping Lin, Jian Guo, Shuwen Deng, Wenjuan Wu, Gustavo H. Goldman, Ling Lu, Yuanwei Zhang

**Affiliations:** 1 Jiangsu Key Laboratory for Microbes and Functional Genomics, Jiangsu Engineering and Technology Research Centre for Microbiology, College of Life Sciences, Nanjing Normal University, Nanjing, China; 2 Department of Laboratory Medicine, Shanghai East Hospital, Tongji University School of Medicine, Shanghai, China; 3 The People’s Hospital of SND (Suzhou New District), Suzhou, China; 4 Faculdade de Ciências Farmacêuticas de Ribeirão Preto, Universidade de São Paulo, Ribeirão Preto, Brazil; Karlsruhe Institute of Technology (KIT), Karlsruhe, Germany

**Keywords:** *Aspergillus fumigatus*, azole resistance, fitness cost

## Abstract

Azoles are the primary antifungal drugs used to treat infections caused by *Aspergillus fumigatus*. However, the emergence of azole resistance in *A. fumigatus* has become a global health concern despite the low proportion of resistant isolates in natural populations. In bacteria, antibiotic resistance incurs a fitness cost that renders strains less competitive in the absence of antibiotics. Consequently, fitness cost is a key determinant of the spread of resistant mutations. However, the cost of azole resistance and its underlying causes in *A. fumigatus* remain poorly understood. In this observation, we revealed that the 10 out of 15 screened azole-resistant isolates, which possessed the most common azole-targeted *cyp51A* mutations, particularly the presence of tandem repeats in the promoter region, exhibit fitness cost when competing with the susceptible isolates in azole-free environments. These results suggest that fitness cost may significantly influence the dynamics of azole resistance, which ultimately contributes to the low prevalence of azole-resistant *A. fumigatus* isolates in the environment and clinic. By constructing *in situ cyp51A* mutations in a parental azole-susceptible strain and reintroducing the wild-type *cyp51A* gene into the azole-resistant strains, we demonstrated that fitness cost is not directly dependent on *cyp51A* mutations but is instead associated with the evolution of variable mutations related to conidial germination or other unknown development-related processes. Importantly, our observations unexpectedly revealed that some azole-resistant isolates showed no detectable fitness cost, and some even exhibited significantly increased competitive fitness in azole-free environments, highlighting the potential risk associated with the prevalence of these isolates.

**IMPORTANCE:**

Azole resistance in the human fungal pathogen *Aspergillus fumigatus* presents a global public health challenge. Understanding the epidemic trends and evolutionary patterns of azole resistance is critical to prevent and control the spread of azole-resistant isolates. The primary cause is the mutation of the drug target 14α-sterol-demethylase Cyp51A, yet its impact on competitive ability remains uncertain. Our competition assays revealed a diverse range of fitness outcomes for environmental and clinical *cyp51A*-mutated isolates. We have shown that this fitness cost is not reliant on *cyp51A* mutations but might be linked to unknown mutations induced by stress conditions. Among these isolates, the majority displayed fitness costs, while a few displayed enhanced competitive ability, which may have a potential risk of spread and the need to closely monitor these isolates. Our observation reveals the variation in fitness costs among azole-resistant isolates of *A. fumigatus*, highlighting the significant role of fitness cost in the spread of resistant strains.

## OBSERVATION

*Aspergillus fumigatus* is an opportunistic fungal pathogen capable of causing a diverse spectrum of acute and chronic life-threatening infections in immunocompromised individuals (1–3). Infections caused by *A. fumigatus* are currently treated with azoles as the first line of treatment, which prevent ergosterol biosynthesis by targeting lanosterol 14-α-demethylase Cyp51A (4). Azole-resistant *A. fumigatus* isolates lead to a substantial burden of treatment failure and pose a grave threat to public health worldwide (5, 6). One of the major mechanisms of resistance is associated with mutations in the *cyp51A* gene that involve tandem repeats (TR) in the promoter region in combination with point mutations in the coding sequence of the *cyp51A* gene (7, 8). Among these mutations, TR_34_/L98H, which confers pan-azole resistance, is the most prevalent type found in clinical settings and environments (9–11). Drug-resistant isolates gain advantages over susceptible isolates in the presence of azole pressure. However, mutants that confer resistance tend to come with a fitness cost, and this fitness cost becomes a disadvantage in the absence of drug-selective pressure (12–14). Therefore, fitness plays a crucial role in determining the rate at which drug-resistant isolates spread in natural populations (15–17). Understanding the fitness cost of azole-resistant *A. fumigatus* provides valuable insights into the distribution of drug-resistant populations and their tendencies to expand in terms of frequency and geographical range.

### Azole-resistant *A. fumigatus* isolates exhibit varied competitive fitness *in vitro*

**Fig 1:**
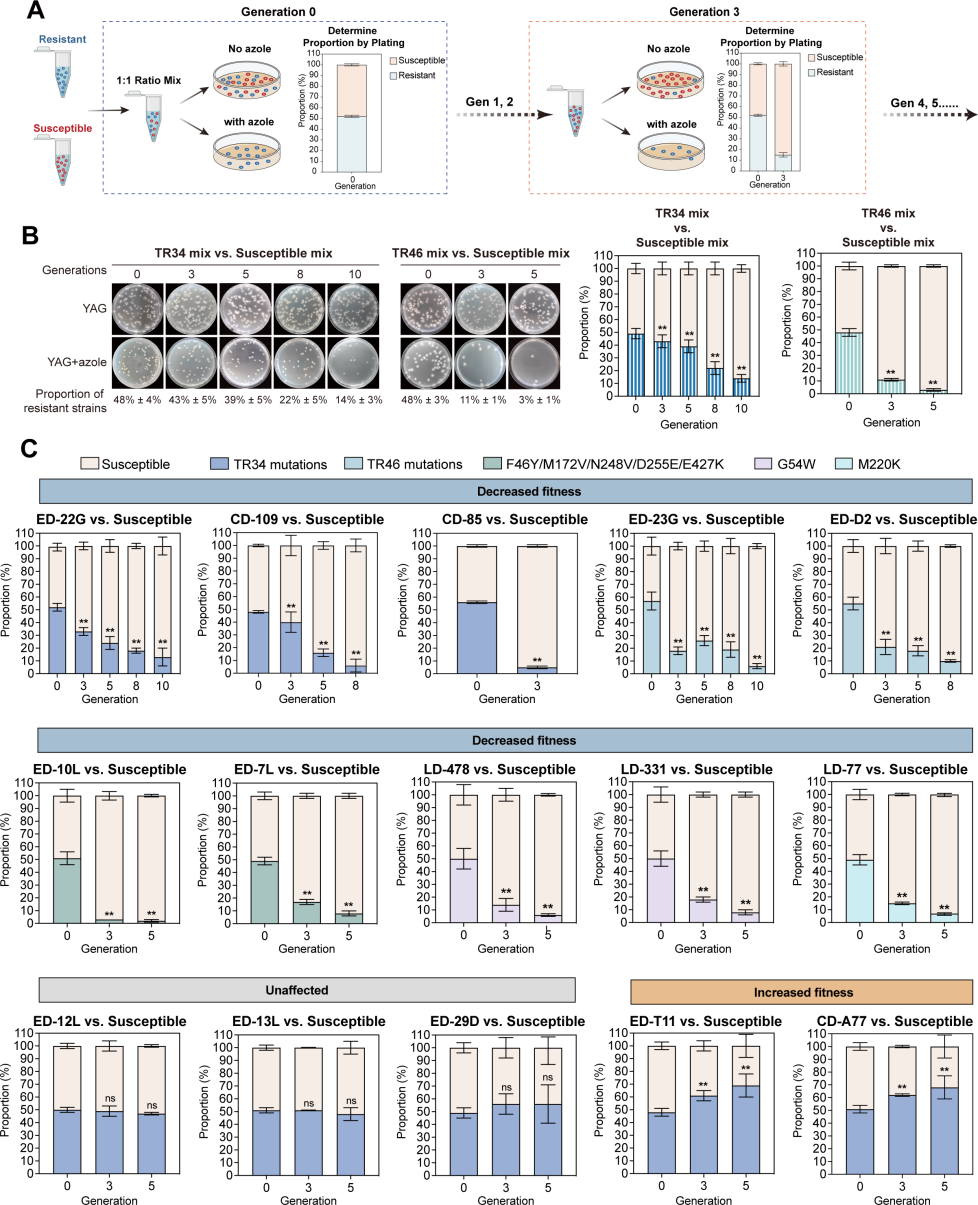
Competition experiments reveal varied competitive fitness among the isolated *cyp51A* mutants. (**A**) Schematic of the *in vitro* fitness assay by colony-forming unit (CFU) counts. (**B**) A mixture of multiple susceptible isolates (nine strains) was used as the susceptible group, which was compared with the TR_34_ mutant group (eight strains) and TR_46_ mutant group (16 strains) for *in vitro* competition. Experiments were performed at least in triplicate with each bar representing the mean ± standard deviation (SD). Statistical analysis was performed using one-way analysis of variance (ANOVA) with multiple comparisons tests. **P < 0.01. (**C**) *In vitro* fitness assay of environment-derived (ED), clinical-derived (CD), and laboratory-derived (LD) azole-resistant isolates. The resistant and susceptible isolates were mixed in a 1:1 ratio, and the culture was transferred every 48 h at 37°C. Two susceptible strains, used for competing with ED and CD isolates, are randomly selected from the environment and clinic, respectively. The susceptible strains used for competing with LD isolates is commonly used laboratory background strain Δ*akuB^KU80^*. The proportion of resistant isolates was counted after 3rd, 5th, 8th, and 10th transfers. Experiments were performed at least in triplicate with each bar representing the mean ± SD. Statistical analysis was performed using one-way ANOVA with multiple comparisons tests. **P < 0.01; ns, not significant.

TRs coupled with point mutations in the *cyp51A* gene are considered as the most common resistance mechanism (18). We therefore sought to dissect the impact of different types of TR repeat mutations on fitness. We first compared the colony growth and conidiation of the azole-resistant isolates with *cyp51A* mutations in solid minimal media and excluded those isolates with apparent morphological defects, which tended to harbor fitness costs when competing with susceptible isolates. To evaluate the overall trends of competitive fitness among azole-resistant isolates, we initially mixed eight isolates with TR_34_ mutations and 16 isolates with TR_46_ mutations, all showing no obvious colony defects, separately. Then, we compared these two mixtures (TR_34_ mix and TR_46_ mix) with a mixture of randomly selected susceptible isolates, all of which were obtained from different independent environmental and clinical sources (Fig. 1B). A competitive assay was employed to measure fitness *in vitro* (15, 19). In this assay, the resistant isolates and susceptible strains were mixed at a 1:1 ratio and cocultured on solid media for 2 days. Then, total conidia were collected and plated onto media in the presence or absence of azole to determine the proportion of the resistant isolates and susceptible isolates (Fig. 1A). As shown in Fig. 1B, both mixed TR_34_ and TR_46_ isolates displayed significantly decreased competitive fitness when competing with the azole-susceptible isolates. These results suggested that azole-resistant *A. fumigatus* isolates from the environment and clinic may harbor fitness costs. However, it cannot rule out the possibility that at least one susceptible strain in the mixture of susceptible isolates may be more fit than the resistant strains and eventually dominate at the end of the competition assay.

To further investigate the factors contributing to fitness cost, 15 resistant isolates with no colony growth defects obtained from environment-, clinic-, and laboratory azole-induced conditions were selected for one-to-one competition assay against corresponding individual susceptible isolates *in vitro* (Fig. 1C; Fig. S1). These isolates contain different *cyp51A* mutations including TR_34_ mutations (TR_34_/L98H and TR_34_/L98H/S297T/F495I), TR_46_ mutations (TR_46_/Y121F/T289A), and point mutations (G54W and M220K and F46Y/M172V/N248V/D255E/E427K) (Table S1). A total of 10 out of the 15 azole-resistant environmental isolates showed a significant reduction in colony-forming units (CFU) when cultured in azole-containing media over generations (Fig. 1B; Fig. S3), indicating that the 10 out of 15 resistant isolates exhibited a significant fitness cost when competing with the corresponding azole-susceptible isolates. Interestingly, we found that three TR_34_ mutations ED-12L, ED-13L, and ED-29D did not manifest any discernible fitness cost despite harboring the same mutations as isolates ED-22G and CD-109, which did have a fitness cost. Notably, two isolates ED-T11 and CD-A77 with TR_34_ mutations even exhibited increased fitness compared to the susceptible strains (Fig. 1C). Together, these results suggested that azole-resistant isolates exhibited varied competitive fitness *in vitro*.

### The competitive fitness is independent of *cyp51A* mutations but might be associated with mutations related to germination rates induced by long-term azole conditions

**Fig 2:**
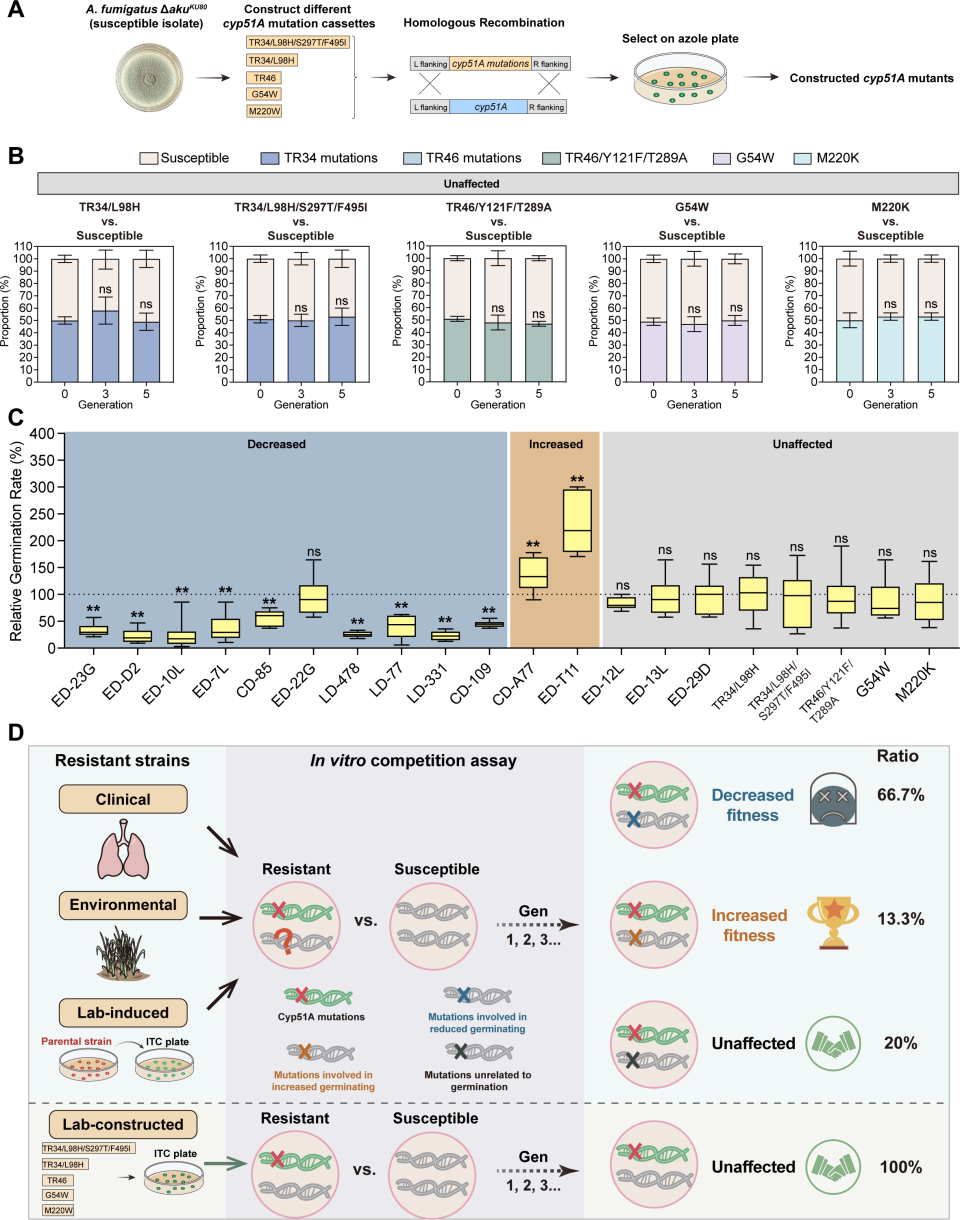
The competitive fitness is not influenced by *cyp51A* mutations. (**A**) Schematic of the construction of *in situ cyp51A* mutation isolates. (**B**) *In vitro* fitness assay of laboratory-constructed azole-resistant *cyp51A* mutants with susceptible parental strain Δ*akuB^KU80^*. A 1:1 mixture of the *cyp51A* mutants and parental strain Δ*akuB^KU80^* was subjected to competition assay at 37°C, with transfers every 48 h and tested for resistance ratios in the 3rd and 5th generations. Experiments were performed at least in triplicate with each bar representing the mean ± SD. Statistical analysis was performed using one-way ANOVA with multiple comparisons tests. ns, no significant. (**C**) Relative germination rate of the azole-resistant isolates compared to the corresponding susceptible strains after 7 h of culture in liquid minimal media at 37°C. The germination rate of the susceptible control strains was set to 100%. Experiments were performed at least in triplicate with each bar representing the mean ± SD. Statistical analysis was performed using one-way ANOVA with multiple comparisons tests. **, *P* < 0.01; ns, no significant. (**D**) Schematic diagram showing a model of variability in competitive fitness among azole-resistant *A. fumigatus* isolates.

To further explore the potential relationship between mutations in the *cyp51A* gene and altered competitive fitness, we constructed *in situ cyp51A* mutations in the triazole-susceptible *A. fumigatus* reference strain Δ*akuB^KU80^* by homologous recombination in a marker-free manner. These mutations included TR repeat mutations TR_34_/L98H, TR_34_/L98H/S297T/F495I, and TR_46_/Y121F/T289A and point mutations G54W and M220K (Fig. 2A). Phenotypic characterization indicated that laboratory-constructed azole-induced resistant *A. fumigatus* isolates exhibited no phenotypic defects (Fig. S2). The competitive assays showed that all laboratory-constructed *cyp51A* mutants did not show any fitness cost (Fig. 2B and C; Fig. S3) when competing with the parental strain Δ*akuB^KU80^*. In addition, we reintroduced the wild-type *cyp51A* gene into the two azole-resistant isolates from the environment (ED-T11) and clinic (CD-109), respectively. The resultant strains were then competed with the corresponding parental azole-resistant isolates, showing that there was no fitness cost for the resulting strains (Fig. S4). These results indicated that the competitive fitness is independent of *cyp51A* mutations. Interestingly, although the colony growth and conidiation of the azole-resistant isolates induced from the environment, clinic, and laboratory were similar to those of the susceptible isolates, we observed notable differences in the conidial germination rates between the resistant and susceptible isolates (Fig. 2C). Specifically, we found that 10 out of 11 resistant isolates with decreased fitness displayed a slower germination rate compared to the susceptible strains, while those with increased fitness exhibited a faster germination rate than that of susceptible strains. Accordingly, those resistant isolates with no significant change in fitness displayed no notable difference in germination rates when compared to the susceptible strains. Collectively, these results suggested that mutations in the *cyp51A* gene are not associated with fitness costs. Instead, we concluded that the occurrence of fitness cost in naturally isolated resistant isolates might be associated with mutations that affected conidial germination rates or other unknown development-related processes during long-term azole treatment (Fig. 2D).

In summary, we comprehensively evaluated the competitive fitness of environmental, clinical, and laboratory-induced *A. fumigatus cyp51A* mutants *in vitro* (Fig. 2D). These mutants exhibited no apparent colony growth defects, yet we observed distinct fitness outcomes among these isolates. Although most isolates showed defective fitness (66.7%), we observed that some resistant isolates exhibited no fitness cost (20%), suggesting that the development of azole resistance in *A. fumigatus* does not necessarily incur a fitness cost or that the presence of compensatory mechanisms mitigate the fitness cost during evolution (Fig. 1C). On the other hand, azole-resistant isolates with higher competitive fitness (13.3%) are intriguing and deserve further special attention due to the high risk for the rapid spread of resistant strains (Fig. 1C). Interestingly, the introduction of *cyp51A* mutations into an isogenic background and reintroduction of the wild-type *cyp51A* gene into the azole-resistant isolates did not yield any fitness cost, implying that *cyp51A* mutations are not directly associated with fitness costs (Fig. 2A and B; Fig. S4). These results were consistent with the previous fitness study using barcode DNA sequencing analysis (20). Further analysis revealed that it is possible that the fitness costs observed in environmental and clinical isolates may be attributed to alterations in other genes related to conidial germination during long-term evolution that are independent of *cyp51A* (Fig. 2C). Our observation reveals the variation in competitive fitness among azole-resistant *A. fumigatus* isolates from the environment and clinic, emphasizing the significant role of fitness ability in dissemination of resistant isolates. Further investigation is needed to unravel the mechanisms underlying these observations.

## Data Availability

The complete data set is presented in the text and the supplemental material.

## SUPPLEMENTAL MATERIAL

Supplemental material mbio.00263-24-s0001.docx Fig. S1–S4, Supplemental Text, and Tables S1 and S2.

ASM does not own the copyrights to Supplemental Material that may be linked to, or accessed through, an article. The authors have granted ASM a non-exclusive, world-wide license to publish the Supplemental Material files. Please contact the corresponding author directly for reuse.
