# Supplementary material for: Variability in competitive fitness among environmental and clinical azole-resistant Aspergillus fumigatus isolates
Source: mBio. 2024 Feb 26;15(4):e00263-24. doi: 10.1128/mbio.00263-24 (PMC11005360; doi:10.1128/mbio.00263-24)
Supplement: Supplemental material — Fig. S1–S4, Supplemental Text, and Tables S1 and S2. [file mbio.00263-24-s0001.docx]

**Supplemental Material**

**
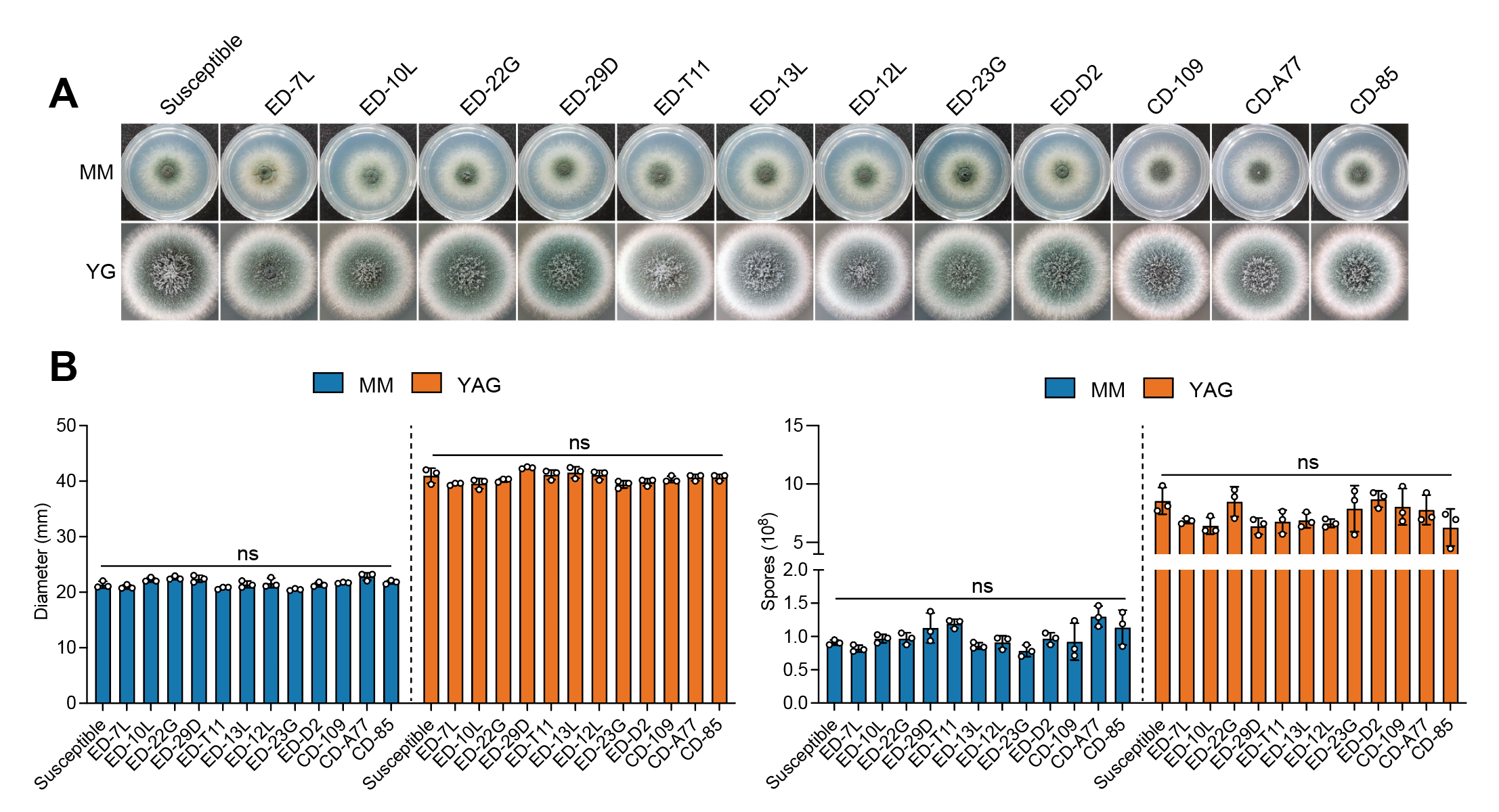
**

**Figure S1. Growth of resistant and susceptible isolates from environment and clinic.**

**(A)** Phenotypes of colonies of resistant and susceptible strains from environment (ED) and clinic (CD) cultured in MM and YAG media at 37°C for 48 h.

**(B)** Comparison of colony diameters and sporulation of environmental resistant and susceptible isolates on MM and YAG media. Experiments were performed in triplicate with each bar representing the mean ± SD, Statistical analysis was performed using one-way ANOVA with multiple comparisons tests. ns, not significant.

**
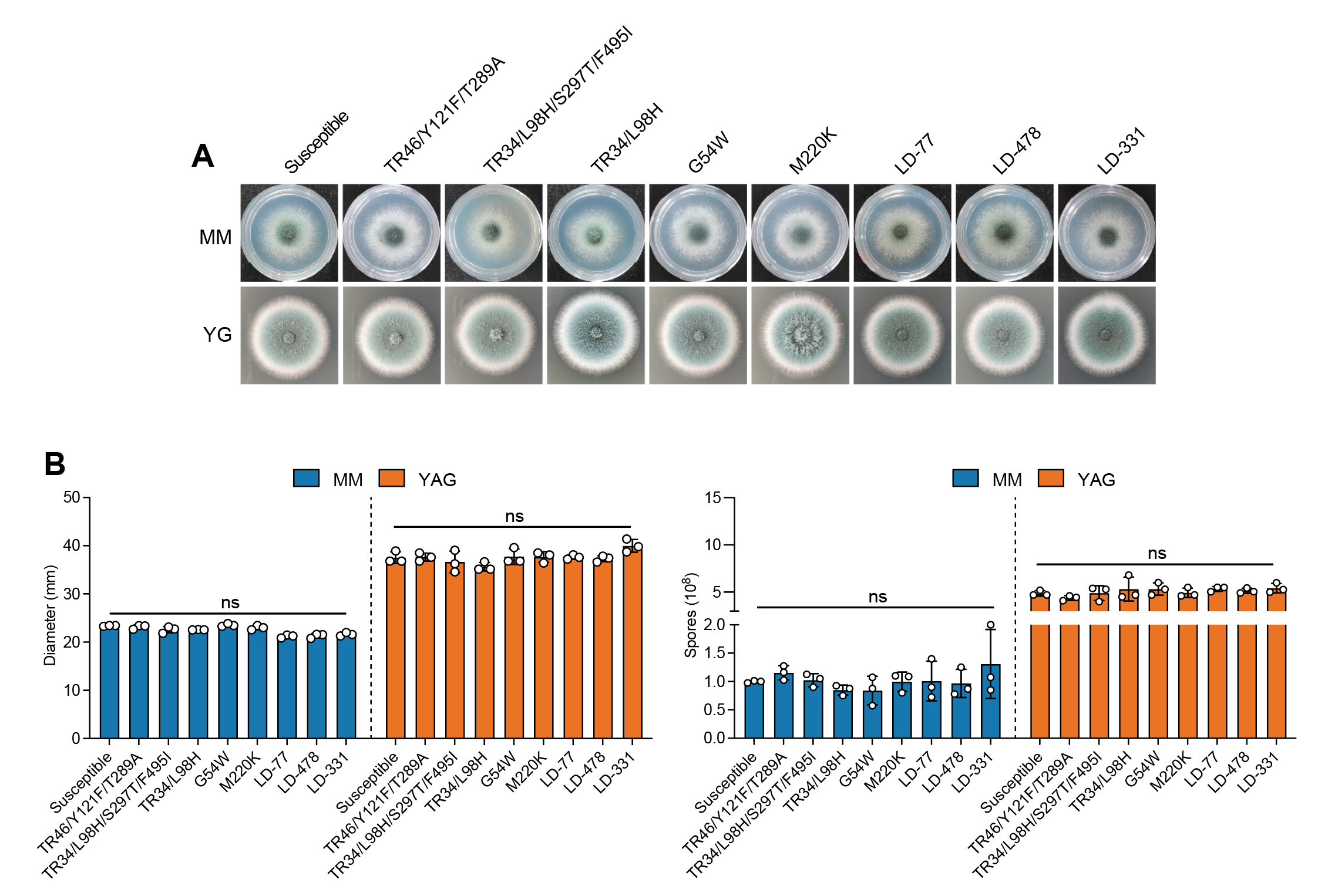
**

**Figure S2. Growth of laboratory constructed and induced *cyp51A* mutants.**

**(A)** Colony phenotypes from laboratory-constructed and induced *cyp51A* mutant and susceptible strains cultured on MM and YAG media at 37°C for 48 h.

**(B)** Comparison of colony diameter and sporulation of laboratory-constructed *cyp51A* mutants and susceptible strains on MM and YAG media. Experiments were performed in triplicate with each bar representing the mean ± SD. Statistical analysis was performed using one-way ANOVA with multiple comparisons tests. ns, not significant.

**
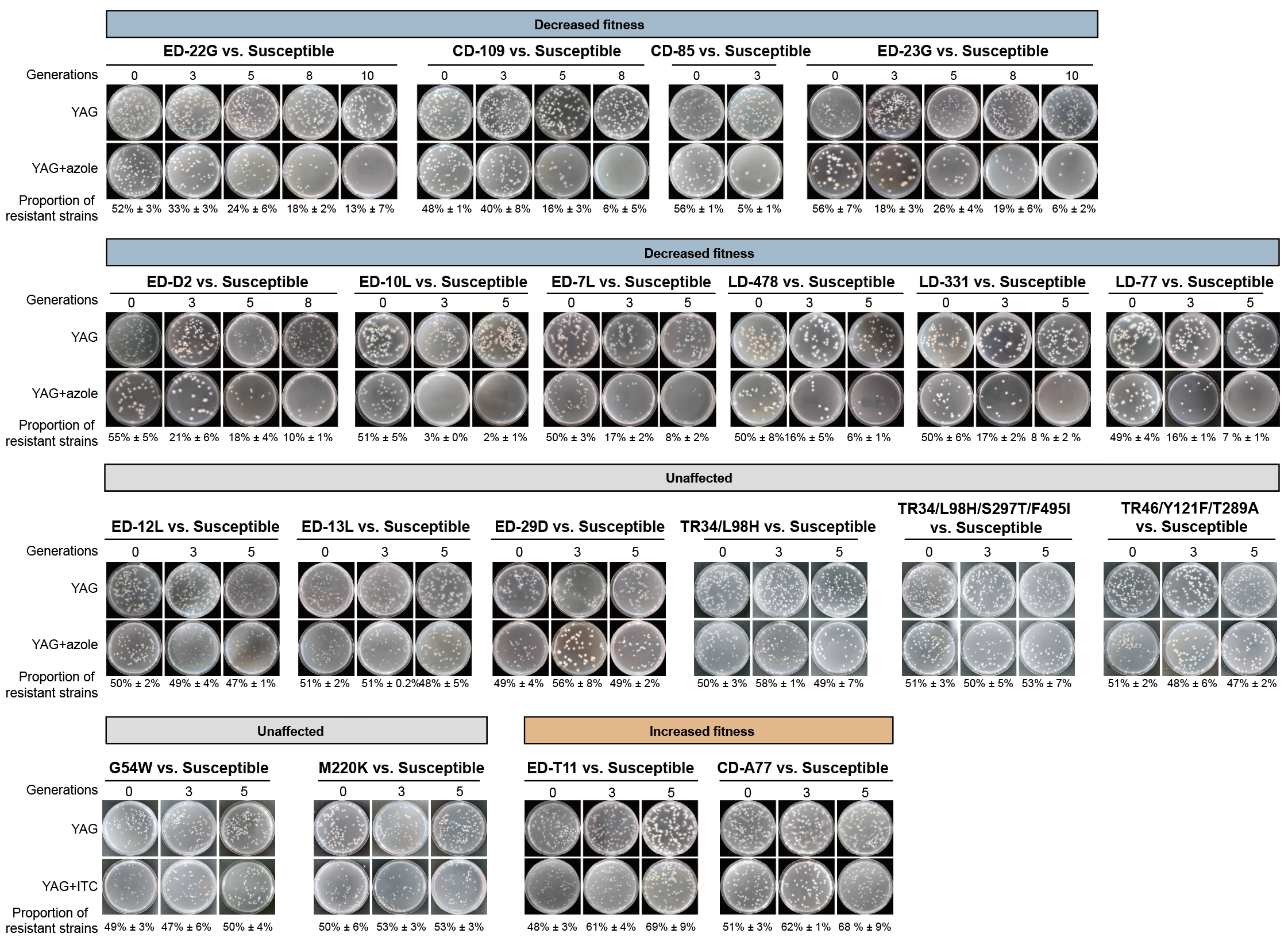
**

**Figure S3. *In vitro* competitive fitness of different types of azole-resistant from environmental and clinical sources.**

Azole-resistant and susceptible strains were mixed in equal proportions and transferred for culture every 48 h under 37°C, and tests were performed on drug and drug-free plates after 3rd, 5th, 8th, and 10th transfers, respectively, and the proportion of resistant strains was counted.

**
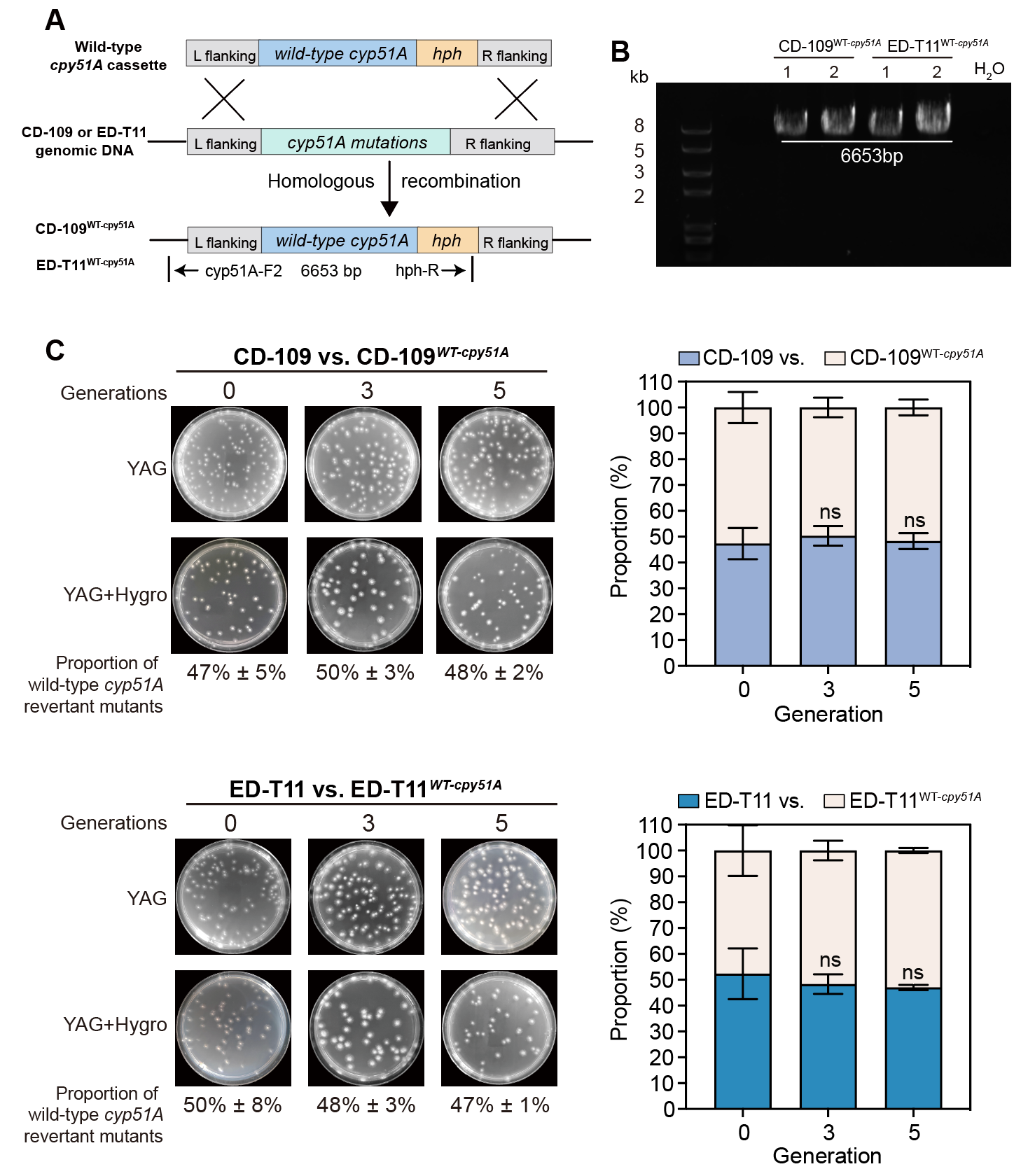
**

**Figure S4. *In vitro* competitive fitness of** **ED-T11 and CD-109 isolates and their wild-type *cyp51A* revertant mutants.**

**(A)** Diagram illustrating the strategy for construction of the ED-T11^WT-^*^cyp51A^* and CD-109^WT-^*^cyp51A^* mutants.

**(B)** Diagnostic PCR using the primers cyp51A-F2 and hph-R confirmed the homologous integration of wild-type *cyp51A* gene in the background of ED-T11 and CD-109 strains.

**(C)** The ED-T11 and CD-109 isolates and their wild-type *cyp51A* revertant mutants were mixed in a 1:1 ratio, respectively, and the culture was transferred every 48 h at 37°C. The proportion of wild-type *cyp51A* revertant mutants was counted after 3rd transfers using 200 μg/mL hygromycin B for selection. Experiments were performed at least in triplicate with each bar representing the mean ± SD. Statistical analysis was performed using two-tailed unpaired Student’s t-test. ns, not significant.

**Materials and methods**

**Strains and growth conditions**

The sources and genotypes of the strains used in this study are listed in Table 1. *A. fumigatus* strains were typically grown on rich medium (YAG: 2% glucose, 0.5% yeast extract and trace elements, 2% agar). Mycelial diameter and sporulation were counted on minimal medium (MM: 1% glucose, 0.1% trace elements and 5% 20 × salt solution 2% agar, pH 6.5). All strains were incubated at 37°C.

**Antifungal drug susceptibility testing**

Itraconazole and voriconazole were tested for susceptibility using a broth-based microdilution method according to the European Committee for Antimicrobial Susceptibility Testing (EUCAST) E.Def. 9.4 method (1).

***In vitro* competition experiments**

The *A. fumigatus* strains were grown on YAG medium at 37°C for 48 hours. Conidia were collected and resuspended in an aqueous solution of 0.05% Tween 20. Azole-resistant environmental isolates and clinical isolates were mixed with azole-susceptible environmental and clinical isolates in a 1:1 ratio, respectively. Similarly, laboratory-induced strains or laboratory-constructed *cyp51A* mutants was mixed with the parental strain in a 1:1 ratio, and 100 μL of the conidia mixture (10^3^ conidia/mL) was evenly spread on YAG plates with or without azole to determine the proportion of resistant isolates. Meanwhile, 100 μL of 10^5^ conidia/mL of the conidia mixture was plated onto the YAG plate and incubated for 48 h at 37°C for next generation. 4 μg/mL Voriconazole was employed to select resistant isolates with TR_46_ mutations, while 4 μg/mL itraconazole was used to select resistant isolates with TR_34_, M220K, and G54W mutations. Competition assay in ED-T11 and CD-109 isolates with their wild-type *cyp51A* revertant mutants were the same as those described above, with the exception of using 200 μg/mL hygromycin B for CFU counting.

**Strain constructions**

All primers used in this study are listed in Table S2. To obtain the *cyp51A* mutation cassettes, the full-length coding sequences of mutated *cyp51A* were amplified using primers cyp51A-F1/R1 from gDNA templates of azole-resistant strains with different mutation types (TR_34_/L98H/S297T/F495I, TR_46_/Y121F/T289A, TR_34_/L98H, G54W, M220K). The wild-type *cyp51A* gene in the background of *A. fumigatus* strain Δ*akuB^KU80^* was replaced with the different mutated *cyp51A* by homologous recombination, the transformants were screened by medium containing 8 μg/mL azoles. The *cyp51A* genes were amplified using the primers cyp51A-F2/R2 and sent for DNA sequencing using the primers cyp51A-P1/P2/P3/R2.

To obtain ED-T11^WT-^*^cyp51A^* and CD-109^WT-^*^cyp51A^* strains, the primers cyp51AWT-P2/P3 and cyp51AWT-P4/P5 were utilized to amplify the left and right homologous arms of Cyp51A using the genomic DNA of wild-type Δ*akuB^KU80^* as a template, and a fragment of the hygromycin B phosphotransferase (*hph*) resistance gene was obtained by amplification with the plasmid pAN7-1 through the primers hph-F and hph-R. The above three PCR products were used as templates for fusion amplification with primers cyp51AWT-P2/P5, and the fusion fragments were introduced into strains ED-T11 and CD-109 by protoplast transformation, and the transformants were screened by medium containing 200 μg/mL hygromycin B. The *cyp51A* genes were amplified using the primers cyp51A-F2/R2 and sent for DNA sequencing using the primers cyp51A-P1/P2/P3/R2.

**Mycelial growth and conidiation evaluation**

Mycelial growth and conidia production were determined on YAG and MM plates. The conidial suspension was diluted to 10^7^ conidia/mL, 2 μL of conidial suspension was added to the centre of the medium. Three plates for each condition and incubated at 37°C for 2 days. The colony diameter was determined by calculating the average of the two vertical directions. Afterwards, all conidia were collected and quantified with a hemacytometer.

**Germination rate test**

Germination was assessed in liquid MM medium with 500 µL of 5 ×10^5^ fresh conidia in 24-well plates. The cultures were incubated at 37°C for 7 h in the stationary phase. Germination of conidia was considered when the length of the germ tube was nearly equal to the diameter of the conidium. The germination rate was determined by quantifying at least three biological replicates of 100 conidia for each strain.

**Table S1. *A. fumigatus* strains used in this study**

| **Strain** | **Source** | **MIC (μg/mL)** | | | **Genotype of *cyp51A*** |
| --- | --- | --- | --- | --- | --- |
|  |  | **ITR** | | **VOR** |  |
| LD-77 | Laboratory induced | >16 | 1 | | M220K |
| LD-478 | Laboratory induced | >16 | 0.25 | | G54W |
| LD-331 | Laboratory induced | >16 | 0.25 | | G54W |
| G54W | Laboratory-constructed | >16 | 0.25 | | G54W |
| M220K | Laboratory-constructed | >16 | 1 | | M220K |
| TR_34_/L98H/S297T/F495I | Laboratory-constructed | >16 | 2 | | TR_34_/L98H/S297T/F495I |
| TR_46_/Y121F/T289A | Laboratory-constructed | 4 | 16 | | TR_46_/Y121F/T289A |
| TR_34_/L98H | Laboratory-constructed | >16 | 8 | | TR_34_/L98H |
| ED-7L | Environment | 2 | >16 | | F46Y/M172V/N248V/D255E/E427K |
| ED-10L | Environment | 2 | >16 | | F46Y/M172V/N248V/D255E/E427K |
| ED-22G | Environment | >16 | 4 | | TR_34_/L98H/S297T/F495I |
| ED-29D | Environment | >16 | 2 | | TR_34_/L98H/S297T/F495I |
| ED-T11 | Environment | >16 | 8 | | TR_34_/L98H/S297T/F495I |
| ED-13L | Environment | >16 | 4 | | TR_34_/L98H/S297T/F496I |
| ED-12L | Environment | >16 | 2 | | TR_34_/L98H/S297T/F497I |
| ED-23G | Environment | 2 | 16 | | TR_46_/Y121F/T289A |
| ED-D2 | Environment | 2 | 16 | | TR_46_/Y121F/T289A |
| CD-109 | Clinic | >16 | 1 | | TR_34_/L98H/S297T/F495I |
| CD-85 | Clinic | >16 | 8 | | TR_34_/L98H |
| CD-A77 | Clinic | >16 | 4 | | TR_34_/L98H |
| D2-2S | Environment | 0.5 | 0.5 | | wild type |
| EC21287 | Clinic | 0.25 | 0.25 | | wild type |

**Table S2.** Primers used in this study

| **Primer** | **Sequence (5’-3’)** |
| --- | --- |
| cyp51A-F1 | CGTACACTGGCAACAGTCA |
| cyp51A-R1 | CGTGATGCAACCATCTCAAT |
| cyp51A-F2 | AGCGAAGATTCCACACATG |
| cyp51A-R2 | GAGCAGGACTGACGAGT |
| cyp51A-P1 | CAGTATGAATTTCTCTGGATGCA |
| cyp51A-P2 | TGTGCTGAGCCGAATGAA |
| cyp51A-P3 | TTCTCAACGGCAAGCTCA |
| cyp51AWT-P2 | GACAACTTGATATCTCGGGCA |
| cyp51AWT-P3 | TCCGTCCGTCTCTCCGCATGCGCTAGTGCTAAGAACTATACATCCC |
| cyp51AWT-P4 | TGCTCCTCTTCTTTACTCTGAACGCAGAGACCAGCGTAT |
| cyp51AWT-P5 | GCCATTCGAACACATTGCA |
| hph-F | GCATGCGGAGAGACGGACG |
| hph-R | TCAGAGTAAAGAAGAGGAGC |

1. Arendrup MC, Friberg N, Mares M, Kahlmeter G, Meletiadis J, Guinea J, Sus SA. 2020. How to interpret MICs of antifungal compounds according to the revised clinical breakpoints v. 10.0 European committee on antimicrobial susceptibility testing (EUCAST). Clin Microbiol Infec 26:1464-1472.
